# Exploring child engagement in a multi-robot tutor-peer learning scenario

**DOI:** 10.3389/frobt.2026.1768482

**Published:** 2026-05-18

**Authors:** Luca Raggioli, Rahul Singh Maharjan, Joanna Kolak, Gemma Taylor, Amanda Banks Gatenby, Angelo Cangelosi

**Affiliations:** 1 Department of Neuroscience, and Reproductive and Odontostomatological Sciences, University of Naples Federico II, Naples, Italy; 2 Manchester Centre for Robotics and AI, University of Manchester, Manchester, United Kingdom; 3 Department of Psychology and Human Development, Institute of Education, University College London, London, United Kingdom; 4 Department of Psychology, University of Salford, Salford, United Kingdom; 5 Manchester Institute of Education, University of Manchester, Manchester, United Kingdom

**Keywords:** child-robot interaction, social robots, multi-robot systems, early language learning, child engagement

## Abstract

**Introduction:**

The use of social robots in education has increasingly focused on how different role framings, such as tutor, peer, or novice, shape children’s engagement and learning. However, most studies employ a single robot whose role is manipulated behaviourally (e.g., the same robot is used to act either as a peer or as a tutor), leaving open the question of how children respond when multiple, physically distinct robots adopt complementary roles within the same session.

**Methods:**

In this exploratory feasibility study, we introduce a blue multi-robot learning environment in which one robot acts as a tutor and the other as a novice peer. The robots supported an early word-learning task in which children learned novel object names and were later asked to name or identify them. In the proposed scenario, participants were exposed to novel words assigned to unusual or unconventional objects (e.g., dog toys and shapes made with interlocking disks), alongside familiar objects. After an initial training phase, children were asked to locate or name the novel items. Sixteen children aged 4–5 years interacted with both robots while their attention, affect, and engagement behaviours were recorded. The study characterised children’s distribution of attention during a word-learning task involving a tutor and a novice peer robot, including attention to task-relevant objects and robots and its relation to attentional complexity, affect, and engagement, as well as the relation between individual characteristics (language ability, age, and media exposure) and task performance, measured as correct naming or pointing during recall.

**Results:**

Children consistently attended to task-relevant objects throughout the session, with attention distributed across objects and robots in structured patterns. Attentional complexity co-occurred with sustained engagement and positive affect. Task performance and engagement showed little variation with age or media exposure, while baseline language ability was negatively associated with recall performance.

**Discussion:**

Overall, the findings indicate that a multi-robot learning configuration is feasible and capable of supporting sustained engagement and structured attentional behaviour in young children. While the results are exploratory and limited by sample size, they provide initial evidence that complementary robot roles can be meaningfully integrated within early learning activities and motivate further systematic investigation.

## 1 Introduction

The use of robots to provide technological support in education has recently emerged as a promising way to complement the roles of teachers and tutors. Robots have been applied in personalised education, notably for students with special needs, offering individualised interaction that in some cases has improved both cognitive and emotional learning outcomes (Kennedy et al., 2015b; Wang and Cheung, 2025). Unlike virtual agents on tablets (McEwen and Dube, 2015; Viriyapong and Harfield, 2013; Otterborn et al., 2019), smartphones (Milne et al., 2014; Hull et al., 2017), or computers (Beatty, 2013), physical robots allow learners to engage with the real world (for example, students with autism (de Vaca et al., 2024)). Their utility becomes especially apparent in tasks requiring direct manipulation or physical demonstration, such as modelling movements (Litoiu and Scassellati, 2015) or practising writing (Hood et al., 2015). The ability of a robot to move and intervene dynamically (rather than being static) is a clear advantage in these settings (Zaga et al., 2015). The efficacy of robots in education depends on context: typical studies involve short and specific lessons, often with limited tailoring to individual students (Belpaeme et al., 2018a). Evidence that robots can fulfil a broader tutoring role analogous to a human teacher remains sparse. For example, robots often enhance affective outcomes (motivation, engagement) without always delivering superior learning gains (Huang and Mutlu, 2014).

Robot appearance significantly influences learners’ perception and interaction (Reich-Stiebert and Eyssel, 2015). In educational contexts, humanoid robots (with head, torso, arms, legs) are common: e.g., the NAO robot with its full range of limb motions. Beyond appearance, a robot’s behaviour plays a crucial role in effective interaction. Recognising attention, confusion, engagement, and other non-verbal cues allows the robot to select appropriate interventions that support sustained learning. Emotional and social scaffolding also matter: a robot might recognise when a child needs a break (Ramachandran et al., 2017), encourage help-seeking (Ramachandran et al., 2016), use appropriate gestures (Huang and Mutlu, 2013a), or focus attention via gaze and posture (Huang and Mutlu, 2013b; Saerbeck et al., 2010). The robot’s interaction style can sway outcomes: in high cognitive-load tasks, an authoritarian style may improve performance (Maggi et al., 2021), whereas overly “sociable” robot behaviour may reduce younger children’s information uptake (Kennedy et al., 2015a; Kennedy et al., 2016).

Among the various role framings explored in the literature (teacher/tutor, peer, novice), the peer role has become widely adopted. According to Belpaeme et al. (2018b), children often treat a robot as a peer in long-term interactions. In this role, the robot’s physical limitations are more acceptable. Research by Zaga et al. (2015), Diyas et al. (2016) shows improved performance when children collaborate with a peer robot on challenging tasks. Expectations matter: as Baxter et al. (2017) note, a tutor robot is expected to perform flawlessly, whereas a peer robot may benefit from imperfection and thereby bolster a child’s confidence and engagement. Sociable and personalised robot behaviour tends to correlate with better learning gains, but trade-offs exist. In STEM tasks (e.g., multiplication tables (Konijn and Hoorn, 2020) or prime-number identification (Kennedy et al., 2015a)), highly social robots have sometimes hindered performance compared with more neutral, task-focused behaviour. Thus, role framing must be chosen carefully, depending on the task and learner.

Recent research has increasingly examined the use of social robots in educational settings, highlighting both their potential to support engagement and their limitations when deployed in complex learning environments Belpaeme et al. (2018a); Serholt et al. (2020). At the same time, recent work has cautioned against overly optimistic assumptions about the benefits of social robots in education. Systematic reviews and empirical studies have noted that observed gains are often context-dependent, short-term, and sensitive to task design, interaction style, and learner characteristics (Lampropoulos, 2025; Wang and Cheung, 2025). Others have highlighted challenges related to cognitive load, role clarity, and mismatched social expectations, particularly when robots exhibit highly sociable behaviour or limited adaptive capacity (Serholt et al., 2020). In multi-agent or multi-robot settings, these concerns may be amplified, as learners must coordinate attention across multiple embodied partners, increasing the risk of distraction or fragmented engagement if roles and interaction dynamics are not carefully designed (Xu and Ouyang, 2025). These observations underscore the need for cautious, empirically grounded investigations that prioritise feasibility and behavioural characterisation before claims about learning effectiveness are advanced. While single-robot systems remain dominant, emerging work has begun to explore multi-robot configurations, raising open questions about coordination, role clarity, and cognitive load for learners. These challenges motivate the need for a systematic investigation of how multiple robots can be integrated within a shared learning activity.

Building on this perspective, the present study explores a multi-robot learning scenario in which two social robots adopt complementary pedagogical roles during an early word-learning task. Rather than evaluating learning effectiveness, the study is designed as an exploratory feasibility investigation, aimed at characterising children’s engagement, attention, and affective behaviour within this interaction setting. It is important to note that, in the present study, the predagogical roles of the robots are not experimentally manipulated. Here, we investigate a fixed multi-robot interaction configuration in which two robots enact complementary roles during a vocabulary learning task. The goal of the study is therefore not to evaluate the causal effects of role assignment, but to explore behavioural engagement patterns that emerge in this multi-robot interaction setting. This exploratory perspective allows us to examine how children allocate attention and express affect in the presence of two socially distinct robotic agents. We defined two research questions to examine the effects of internal (i.e., elements manipulated or measured within the experiment) and external (e.g., language proficiency and household composition) factors on children’s engagement and performance:

RQ1: How do children spread their attention during a word-learning task with a tutor and a peer robot? This includes (a) gaze allocation between task-relevant objects and the robots, (b) associations between attentional complexity and attentional focus, and (c) behavioural predictors of positive affect directed toward either the task or the robots. Evaluating the balance between cognitive engagement and disengagement was essential for determining whether the multi-robot configuration supported sustained participation.
RQ2: How are children’s levels of task engagement and performance related to individual characteristics such as language comprehension, media exposure, and household composition? We included age as a factor to determine if developmental differences within the 4–5 years range influenced interaction dynamics. This analysis targets whether baseline language ability, age, household composition, and digital media exposure are associated with children’s engagement behaviour or learning outcomes, serving as a check on the robustness of the multi-robot approach across this demographic.

The remainder of this paper is structured as follows: Section 2 reviews relevant literature, highlighting both opportunities and challenges associated with deploying multiple social robots in learning contexts. details the experimental design and behavioural coding methodology used to characterise children’s attention, affect, and engagement. Section 4 presents the results, reporting the observed patterns of behaviour and performance. Finally, Sections 5, 6 discuss the implications and limitations of these findings for future multi-robot educational research.

## 2 Background and related work

### 2.1 Role of robots in the educational scenario

The strategy by which the robot interacts with the student plays a crucial role in successful interaction and learning processes. There are three main roles that the system can assume (Belpaeme et al., 2018a; Van den Berghe et al., 2019): the *Robot as a Tutor*, the *Robot as a Peer*, and the *Novice Robot*.

In the robot tutor strategy, the system delivers key elements of frontal lessons, overseeing students, providing explanatory examples, and offering suggestions to facilitate concept learning (Belpaeme et al., 2018a; Saerbeck et al., 2010). When the robot acts as a peer, it positions itself at the student’s level, acting as a classmate and collaboratively supporting the learning process. This approach can be beneficial as it might be less intimidating than interacting with a teacher (Zaga et al., 2015; Baxter et al., 2017). Finally, in the novice robot strategy, it takes on the role of a student in need of help, while the student becomes the tutor. This strategy can be effective in building the student’s confidence and awareness, leading to improved learning outcomes (Tanaka and Matsuzoe, 2012; Hood et al., 2015).

#### 2.1.1 Tutor robots

An example of this strategy is illustrated in You et al. (2006), where a RoboSapien robot is utilised in an English course for children aged 6 to 12 at a Taiwanese school. The robot assumes an assistant teacher role, employing five interaction modes to complement teaching: “Storytelling” to motivate language learning, “Q&A” where the robot randomly asks students English questions, “Cheerleader” with celebratory dances or sounds, “Let’s Act” where the robot mimics student movements upon the teacher’s approval, and “Pronunciation leading” where the robot guides students in English pronunciation. Ramachandran et al. (2018) targets the think-aloud strategy on learning outcomes in a 2 × 2 between-subjects study, where a NAO robot would remind or prompt sixth grade children (approximately 11 years of age) to express their thinking process out loud while solving maths problems, and would instruct them to reflect on why an answer might be incorrect, in the case of wrong answers. Results showed enhanced learning, engagement, and compliance towards a cognitively complex task. More recent work extends the tutor framing: for instance, Konijn and Hoorn (2020) studied primary-school children rehearsing multiplication tables with a social robot tutor (autonomous) and found that ability level and robot feedback mode moderated the effectiveness of the robot as tutor. In another study, Almousa and Alghowinem (2022) developed a personalised robot tutor for preschool children (ages 3–5) that adapted lesson difficulty, gaze, and feedback to the child’s body orientation, hesitation time, and gaze direction, showing promising gains in engagement and knowledge. These examples show that tutor robots are increasingly autonomous and personalised, though they are still often used in short-term, one-on-one sessions rather than in full classroom settings.

Despite promising findings, meta-analytic evidence suggests that tutor robots yield small and heterogeneous learning effects compared to alternative instructional media (van den Berghe, 2022; Belpaeme et al., 2018a). Several comparative studies report no clear advantage over screen-based or tablet-based interventions Vogt et al. (2017). Furthermore, children’s tendency to attribute high competence and authority to embodied agents may foster compliance rather than deep cognitive engagement (Serholt et al., 2020). Consequently, the pedagogical added value of tutor robots remains an open empirical question rather than an established fact.

#### 2.1.2 Peer robots

In Kanda et al. (2004), two Robovie robots were introduced in a Japanese elementary school for 18 days. The robots interacted with students using gestures and English phrases whenever they were approached, and were recognised by wireless tags and various sensors. The results suggested that having common conversation topics could make interactions more interesting and stimulating from both a social and technological standpoint. Kory (2014) employed a peer tutor robot in a storytelling game setting to teach new words to children aged between 2 and 6 years old. The game was played together by a child and the robot, using a tablet, on which the story being constructed had to be filled in by dragging items on the screen. The robot’s behaviour was either adaptive, adjusting the difficulty of its words as the child progressed and according to their spoken language level, or non-adaptive, always using the same (low) level of difficulty. In the adaptive case, children’s diversity of language improved more, suggesting that an adaptive robot keeps the interest and engagement of the child higher, thus leading to better learning. More recently, Chen et al. (2025) found that when children observed a robot peer failing (making errors) while solving a task, the children themselves improved their learning: the peer-robot’s visible mistakes enabled them to reflect more deeply. This supports the idea that peer robots do not simply collaborate but can stimulate meta-cognitive activity by acting imperfectly and revealing that mistakes are part of learning. Peer robots thus can create an environment of joint exploration rather than one of expert instruction.

However, positioning the robot as a peer can blur instructional authority and reduce clarity in task guidance (Zaga et al., 2015). While peer configurations are frequently associated with increased engagement and social presence, learning outcomes are not consistently stronger than in the tutor-based condition (van den Berghe, 2022; Kory-Westlund, 2023). Additionally, children’s interpretation of a robot as a peer may lead to inconsistent expectations, interactional breakdowns, or distraction from task goals (Serholt et al., 2020; Belpaeme et al., 2018a).

#### 2.1.3 Novice robots

Examples of this strategy include using a small humanoid Care-Receiving Robot in a Japanese school during English lessons for children aged three to 6 years (Tanaka and Matsuzoe, 2012). The robot intentionally makes mistakes that the children can correct. Another example involves children teaching the robot correct handwriting (initially, the robot’s handwriting is poor) through demonstration (Hood et al., 2015). Building on this, Pareto et al. (2022) compared children learning-by-teaching a robot tutee versus a younger child tutee, finding that children guiding the robot worked at deeper levels of explanation and reported higher confidence afterwards. Similarly, Tarakli et al. (2025) recently explored a peer-like robot that learned via interactive reinforcement learning and found that children acting as tutors for the robot showed greater retention of knowledge than children practising the content themselves.

Nevertheless, learning-by-teaching approaches with robots introduce additional interactional and cognitive demands for young learners (Hood et al., 2015; Schodde et al., 2017). Children must both articulate explanations and monitor the robot’s behaviour, increasing task complexity relative to direct practice. Furthermore, many studies rely on short-term deployments, leaving questions about long-term sustainability and the influence of novelty effects on engagement and learning.

### 2.2 Embodiment, behaviour and social responsiveness

Physical embodiment gives robots advantages over virtual agents, enabling spatial, gestural, gaze behaviours and richer multimodal interaction (Belpaeme et al., 2018a). The robot’s appearance influences expectations: humanoid robots evoke higher social attributions and potentially greater engagement (Reich-Stiebert and Eyssel, 2015). Robot behaviours, such as attention detection, emotional support, and scaffolding, are critical for effective interaction (Leite et al., 2014; Ramachandran et al., 2017). Very recent work indicates inclusive benefits of multimodal robots for students with special needs (e.g., dyslexia) via dynamic interaction design (Fung et al., 2025). For example, Kim and Tscholl (2021) examined how a physically embodied humanoid robot engaged children aged 3–6 years in play and learning in both home and school settings. They found that embodiment supports richer multimodal interaction, extended attention spans, and voluntary peer collaboration. Another study by Wedenborn et al. (2019) compared a physical robot tutor with a screen-based avatar or voice-only condition in vocabulary learning, reporting significantly higher recall in the physical robot condition, suggesting embodiment directly contributes to learning gains.

### 2.3 Child-robot and multi-robot interaction studies

Research on child-robot interaction (CRI) has increasingly explored how children perceive and collaborate with robots. For example, children’s social relations with a robot (such as rapport, turn-taking, mirroring) have been linked to stronger learning outcomes in vocabulary tasks (Kory et al., 2019). Multi-robot studies are more emergent but show promise: the system “ChildBot” used multiple robotic agents with children and demonstrated improved perception and coordination in a sample of 52 children (Efthymiou et al., 2020). Multi-robot arrangements can facilitate joint attention, social scaffolding, and dynamic role division. Design challenges remain: attention switching, role clarity, cognitive load, and coordination between robots may become burdens rather than benefits if not well structured (Zaga et al., 2015). A recent review by Xu and Ouyang (2025) highlights how multi-agent educational robotics is evolving and indicates the need for clearer role differentiation, synchronisation strategies, and longitudinal studies. To date, few studies have assigned distinct pedagogical roles to two or more robots in the same session (tutor and novice) and measured the combined effect on a single learner’s engagement and learning. Although multi-robot systems are beginning to emerge, most prior work either employs homogeneous robot roles or primarily investigates inter-robot coordination mechanisms rather than the child’s distributed attention and learning processes (Baxter et al., 2017; Bravo and Páez, 2023). Empirical evidence on how a single child interprets, prioritises, and coordinates interaction with two role-differentiated robots within the same learning task remains scarce (Dahiya et al., 2023). Moreover, interacting with multiple embodied agents may increase cognitive and attentional demands, potentially imposing additional processing burdens on young learners (Zaga et al., 2015; Serholt et al., 2020). For instance, Leite et al. (2015) compared disengagement models in individual versus group interactions, highlighting how multi-party dynamics can significantly alter engagement patterns compared to dyadic settings. Empirical studies have also begun to explore learning scenarios involving multiple robots, where distinct robotic platforms jointly participate in instructional activities and influence learner engagement and affective responses during educational tasks (Fung et al., 2025). In parallel, integrated multi-robot systems such as ChildBot demonstrate how heterogeneous robots can collaboratively support interaction with children by combining complementary sensing and expressive capabilities, illustrating the technical motivations for multi-robot configurations in educational environments (Efthymiou et al., 2022).

Across child-robot interaction research, engagement is commonly conceptualised as a multidimensional construct encompassing affective, cognitive, and behavioural components (Leite et al., 2014; Maggi et al., 2025). In learning contexts, affective engagement reflects children’s emotional valence during interaction (e.g., enjoyment or withdrawal), cognitive engagement is often operationalised through attention allocation and attentional dynamics, and behavioural engagement captures embodied indicators such as posture and physical orientation. These dimensions have been shown to relate to learning outcomes and interaction quality in both single-robot and multi-agent settings. Given the social and attentional demands introduced by interacting with two role-differentiated robots, the present study adopts this tripartite framework to characterise how children distribute attention, express affect, and physically orient during the learning task. These constructs informed the video coding scheme described in Section 3.4.

## 3 Methods

### 3.1 Materials

The experimental setup employed two commercially available humanoid robots: Pepper and NAO (see Figure 4a), both developed by SoftBank Robotics. Pepper^1^ is a human-sized humanoid robot (approximately 1.2 m tall) designed for social interaction. It is equipped with a touchscreen, multiple microphones, RGB and depth cameras, and articulated arms and head, enabling expressive gestures, gaze behaviours, and multimodal interaction. NAO^2^ is a smaller humanoid robot (approximately 58 cm tall) with 25 degrees of freedom, allowing for precise body movements, head orientation, and gestural communication. In this study, Pepper was assigned the role of tutor and interaction leader, while NAO was framed as a novice peer, leveraging their differences in size, appearance, and perceived authority.

**FIGURE 1:**
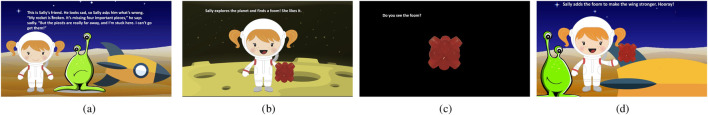
Story used as base for the experiment. The leading avatar introduces the alien friend with the task to solve **(a)** she finds an object that is necessary in the task **(b)** which is also shown without any background **(c)** finally she uses the object, during the testing phase, to complete the task **(d)**. Images reproduced with permission from https://osf.io/nbrhk (Piazza et al., 2021).

The choice of the learning task is a crucial step for designing a successful child-robot interaction session. It is important to use a scenario with a verified educational strategy that can be easily adapted during a robot session. To do so, we need a task where all the word naming occurs in the context of a story that can be narrated by the robot, while being realistic: it is necessary, in fact, that the object fits well in the narrative so that it does not become confusing. The story used for our task is based on the material provided in a storytelling study (Piazza et al., 2021), where novel words and objects are embedded in an unfolding story^3^ (Figure 1). An astronaut’s avatar addresses the reader, stating that she needs to find four items to help her alien friend repair his spaceship and return home. The objects were presented in three distinct phases:

- First word exposure: where the object is found and presented in context.
- Isolated word exposure: where the object is shown on a black background and named again.
- Function word exposure: where the object is presented again, describing how it could be used to fix the spaceship.

The first two exposures are part of the training phase, while the last one is part of the testing phase.

**FIGURE 2:**
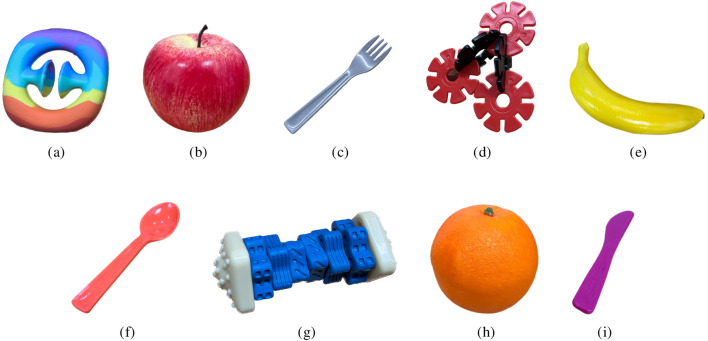
Target objects used in the experimental sessions, coupled with respective competitors: the Modi **(a)** is matched with an apple **(b)** and a fork **(c)** the Koba **(d)** is matched with a banana **(e)** and a spoon **(f)** the Sprock **(g)** is matched with an orange **(h)** and a knife **(i)**.

The structure of our experimental session draws inspiration from the one outlined in Piazza et al. (2021), with the plot also following their story closely. The story revolved around an alien friend of the robot, narrated by the robot itself. The alien had lost some of his toys, needed to fix his spaceship, and wanted to return home. In our case, we use three objects. The reason for this is that we want the objects to be all clearly in the field of view of the child, so not too far away from each other, while still allowing the robot to look at the target in an unambiguous and unmistakable way. In fact, we observed that with four objects in the robot’s field of view, the target of the robot’s gaze was not always clear. The gaze is used in this study as the main robot’s social cue employed. It is important to note that for both the physical objects and their referents, the objective was to use items that would be less likely known by the participants, as in Piazza et al. (2021). Specifically, the objects were chosen by consulting the NOUN dataset^4^, which contains unfamiliar objects (mostly animal toys) and unknown words. We chose the words “*Modi*”, “*Koba*”, and “*Sprock*”, respectively matched with a fidget toy (Figure 2a), a shape obtained with interlocking disks (Figure 2d), and a dog toy (Figure sg). The novel words were chosen empirically based on how clearly the robot’s text-to-speech would utter nouns in the dataset. Each target is matched with two competitors that might be familiar words for the target age group: a fruit (apple, banana, or orange) and a cutlery item (fork, spoon, or knife).

The session is articulated as follows:

1. Introduction: The robot leading the interaction greets the child and introduces the NAO robot. Then, it proceeds to present the problem, asking for help in locating the necessary toys. In the session, we chose to refer to the NAO robot as “*Paprika*” to avoid ambiguity with the English word “now”. The name was chosen to use a friendly and playful name, which, similarly to Pepper, references a spice.
2. Training phase for the three targets:
  - First exposure: a target object is shown together with two competitors (Figure 2). The leading robot says the name of the target it needs to find, looks around, and then turns its head towards it, repeating the novel name.
  - Isolated exposure: after both the leading robot and the child have identified the target, an experimenter removes the competitors. The child is then asked to repeat the new word (after it is again repeated by the robot). The child is also encouraged to pick up the target object to see it more closely.
3. After all the objects have been found, the three targets are presented together to the child, with the robot repeating all three novel names without looking at any of the objects.
4. Testing phase in which the child’s recall of the correct matching is examined. Two different testing conditions were considered:
  - Expressive learning: The robot looks at an object and asks the child to remember the name.
  - Receptive learning: The robot says the name of a target and asks the child to point at it among the objects on the table.

This manipulation was between participants.

5. The session concludes, and the robot thanks the child for the help.

**FIGURE 3:**
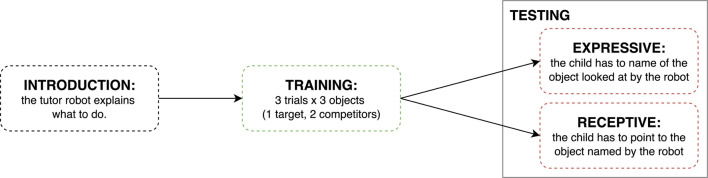
Experimental flow diagram illustrating the sequence of phases: Introduction, Training with three objects, and Testing with the between-subjects condition assignment (Expressive vs. Receptive; for more simplicity in the rest of the manuscript, these are referred to as Naming and Pointing).

To clarify the experimental flow, the sequence of phases is illustrated in Figure 3.

### 3.2 Participants

The study was approved by the Ethical Committee of the University of Manchester. We recruited 16 children aged 4–5 years old ( N=16 , mean age=4.44 , SD=0.51 , 50 % females). Due to the young age of the participants, it was not possible to provide them with a participant information sheet. Before the start of the session, they were asked for verbal assent after the experimenter explained to them what they would be expected to do. Besides the 16 children who completed the experiment, two more children were initially recruited, one male and one female. However, the former had to be excluded due to significant changes in the setting (the room was changed starting with the second child taking part in the study) and malfunctioning of the robot, which stopped receiving the commands and had to be rebooted (effectively disrupting the session). The latter, instead, was initially excited about seeing robots in person, but upon entering the room and seeing the robots turning on, started crying, and we had to cancel the session. The age range was chosen to target children between the end of the reception year and the start of year one of primary school^5^, with the objective of not having too drastic differences in the participants’ language level. Recruitment was conducted from June 2023 to February 2024, and it was significantly impacted by the COVID-19 pandemic, as schools remained resistant to participating in research involving novel technologies. While some schools agreed to share with parents information about the study and the researchers’ contacts, most schools declined this opportunity, claiming that parents of their pupils would not allow participation in such types of activities. In accordance with the University’s Ethical Committee, communications with these schools were interrupted to not cause discomfort. The final participant group was obtained as a convenience sample, recruited through the University of Manchester’s internal mailing lists. All accompanying parents held either a Bachelor’s or a Doctorate/Specialisation level of education. Details about the participants are reported in Table 1.

**TABLE 1:** Family background, digital device ownership, and language exposure.

ID	G	Age	Parents’ degrees	Devices	Sib	Languages exposure
1	M	4	Bachelor or master; doctorate	5	0	3 (English, Mandarin, Tamil)
2	F	5	Bachelor or master; high school	3	1	1 (English)
3	M	4	Doctorate; high school	1	0	2 (English, Italian)
4	F	5	Bachelor or master; not disclosed	2	1	2 (English, Russian)
5	M	5	Both bachelor or master	2	1	2 (English, Malaysian)
6	F	4	Both bachelor or master	1	0	2 (Malay, English)
7	M	4	Both bachelor or master	3	1	2 (Malay, English)
8	F	5	Both bachelor or master	2	1	2 (Malay, English)
9	M	5	Both bachelor or master	4	1	1 (English)
10	M	5	Both doctorate	10	1	1 (English)
11	F	4	Both bachelor or master	5	1	2 (English, Dutch)
12	F	4	Both bachelor or master	4	0	2 (English, French)
13	F	4	Both bachelor or master	5	1	1 (English)
14	M	4	Both bachelor or master	5	1	1 (English)
15	F	4	Both doctorate	6	2	5 (English, Urdu, Punjabi, Arabic, Turkish)
16	M	5	Both bachelor or master	6	2	1 (English)

### 3.3 Procedure

**FIGURE 4:**
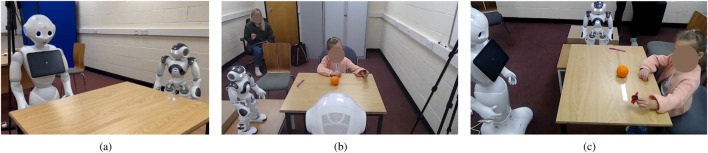
Setting of the Tutor-Peer condition **(a)** where the Pepper is standing across the child, the NAO is standing on a small table to the child’s right, and the three objects are placed on the table **(b)**. The position of the objects is marked on the table with transparent tape so that the experimenter can always place them in the correct place **(c)**.

Upon arrival, parents provided informed consent, which also requested permission for recording videos of the session. This was also reported in the Participants Information Sheet, which was distributed via email during the recruitment. All parents gave consent for their child to take part in the experiments and for videos to be recorded. They also gave consent to fill out a demographic questionnaire, developed by the e-LADDA network^6^, regarding household composition and the child’s digital media exposure. Concurrently, the child participated in a 5-min familiarisation phase, interacting with the robots to become accustomed to them. The experimental session then commenced (Figure 4). Parents typically remained in the room, positioned behind the child to minimise influence, though some sat nearby upon request.

The robots operated autonomously, with an experimenter manually inputting the child’s responses to trigger appropriate behavioural branches. Specifically, the interactions between the two robots were scripted (in order to have the same utterances in every session) with the robot movements associated with the robots’ speech being autonomously generated by the robot API’s Animated Speech module. Whenever the child’s reaction or answer was necessary, the script would remain on hold waiting for a keyboard input entered by the experimenter. For instance, in the starting sequence, the Pepper introduces itself and then the NAO, which waves and greets the child. This exchange runs without any external input. Next, the Pepper asks for the child’s name (which was already codified in the script prior to the start of the session) and waits for the child’s answer. Depending on the child’s answer or refusal to engage, the experimenter triggers a robot’s response, and the script’s execution continues. Pepper acted as a tutor, leading the interaction, while NAO (positioned to the child’s right, as in Figures 4b,c) acted as a novice peer. Specifically, the main purpose was to The tutor occasionally queried the peer; if asked before the child, the peer provided incorrect or “do not know” answers. If asked after, the peer confirmed the correct answer. During training, robot feedback was contingent on the child’s responses. During testing, feedback was neutral to avoid bias.

After the interaction, children completed the Quick Interactive Language Screener (QUILS)^7^ (Golinkoff et al., 2017) on a tablet. The QUILS is a standardised, norm-referenced assessment for children aged 3 through 6 years, evaluating vocabulary, syntax, and language processing skills. Finally, families were thanked and received a £10 voucher and a certificate of participation.

### 3.4 Video coding and behavioural metrics

All interaction sessions were video-recorded and subsequently annotated using *ELAN 7.0* ^8^. Following the framework proposed in Maggi et al. (2025), the coding scheme focused on three broad engagement dimensions (affective, cognitive, and behavioural) to annotate the children’s moment-by-moment responses, and enabled the extraction of fine-grained behavioural measures for quantitative analysis. Specifically, the categories are coded as follows:

- Affective State. Children’s affective engagement was coded continuously, marking visible cues such as smiling, neutral expressions, and negative or withdrawn expressions. Continuous coding refers to exhaustive, non-overlapping temporal segmentation.
- Possible values: *positive, neutral, or negative*.
  These annotations formed the basis for the affective metrics detailed in Table 2. Positive affect overlaps were computed by identifying moments where the child displayed positive affect while gazing at a specific interaction target. All ratios are calculated as the cumulative duration of the behaviour divided by the total session time.
- Gaze Behaviour. Gaze direction was annotated, capturing where the child oriented their attention across the interaction space at all times.
- Possible values: *Pepper, NAO, parent, objects, or elsewhere (e.g., one of the two experimenters or any distracting element in the room)*.
  From these annotations, we derived relative gaze ratios and trajectory metrics quantifying attention shifts between targets (see Table 2). Engagement and disengagement metrics were also aggregated to compare overall gaze attention towards the task.
- Behavioural Engagement. Operationalised as posture and physical orientation, it was coded continuously, distinguishing periods in which the child was leaning forward, leaning back, sitting upright, or leaning laterally.
- Possible values: *sitting, leaning laterally, leaning forward, leaning back, standing up*.
  These were aggregated into the behavioural metrics listed in Table 2.

After annotation, all coded timelines were synchronised and aggregated. A complete summary of all derived metrics, including calculation methods for ratios, trajectory measures, and external factors (language scores, household composition, and technology exposure), is provided in Table 2. Ratios represent the proportion of session time spent in a given state or directing attention to a given target. Trajectory measures quantify the dynamism and complexity of children’s attention shifts. Specifically regarding external factors, the *robot_exposure_ratio* was calculated as a weighted average of questionnaire responses regarding exposure to robots in various media and real life, mapped to a 0–1 scale.

**TABLE 2:** Summary of the behavioural metrics and ratios defined in the coding scheme. All time-based ratios are calculated by dividing the cumulative duration of the behaviour by the total duration of the session.

Category	Metric variable	Calculation method
Affective state	*positive_affect_ratio*	Cumulative duration of positive affect/Total time
	*neutral_affect_ratio*	Cumulative duration of neutral affect/Total time
	*negative_affect_ratio*	Cumulative duration of negative affect/Total time
	*affect_robot_ratio*	Duration of (positive affect ∩ gaze at robots)/Total time
	*affect_task_ratio*	Duration of (positive affect ∩ gaze at objects)/Total time
Gaze behaviour	*pepper_gaze_ratio*	Cumulative duration of gaze at pepper/Total time
	*nao_gaze_ratio*	Cumulative duration of gaze at NAO/Total time
	*objects_gaze_ratio*	Cumulative duration of gaze at objects/Total time
	*cognitive_engagement_ratio*	(Gaze at robots + gaze at objects)/Total time
	*cognitive_disengagement_ratio*	(Total time - cognitive engagement time)/Total time
	*gaze_trajectories*	Average frequency of gaze shifts only involving pepper, NAO
		Or any of the objects (e.g., pepper-NAO-apple-pepper-…)
Behavioural engagement	*sitting_ratio*	Cumulative duration of sitting posture/Total time
	*leanforward_ratio*	Cumulative duration of leaning forward/Total time
	*posture_changes*	Count of posture transitions (count)
Performance	*training_performance*	(0.5 × # pick up + 0.5 × # correct naming)/Num. Of targets
	*test_performance*	Number of correct answers/Number of targets
External factors	*robot_exposure_ratio*	Weighted average of questionnaire responses (scaled 0–1)
	*vocabulary*	Score from QUILS language screener (vocabulary section)
	*syntax*	Score from QUILS language screener (syntax section)
	*process*	Score from QUILS language screener (process section)
	*overall_screening*	Total score from QUILS language screener
	*siblings*	Number of siblings (questionnaire)
	*own_digital_devices*	Child’s ownership/usage of digital devices (questionnaire)

Task performance was assessed by examining both the training and the testing phase. The training performance is obtained as an aggregate estimate considering both if the child picked up the correct object and then if the name of the target was repeated correctly. Test performance was instead calculated as the number of correct naming/pointing divided by the number of targets. Both are shown in Table 2. It is important to note that, while it might seem obvious that children would pick up the intended targets, this did not always happen during all sessions. Some children, in fact, did not initially engage with the robot when asked to locate the target, while another child did not pick up the last object of the training batch. For this reason, this estimate allows us to reward picking up the target object as well as the correct naming association.

### 3.5 Data analysis strategy

In order to address the research questions defined in the Introduction, we employed a combination of non-parametric and parametric statistical methods. Given the small sample size (N=16) , these analyses are intended as descriptive and exploratory rather than confirmatory. They serve to characterise the observed behaviours and identify potential trends to guide future hypotheses. For RQ1, we used Wilcoxon signed-rank tests to compare paired observations (e.g., gaze allocation to objects vs. robots, cognitive engagement vs. cognitive disengagement), and Spearman rank correlations to examine associations between attentional complexity and gaze allocation. Given the small sample size, analyses were limited to non-parametric correlations to explore associations between behavioural measures, individual characteristics, and task performance. For RQ2, we employed Spearman rank correlations to assess relationships between individual characteristics (language ability, age, household composition) and learning outcomes or engagement metrics. All analyses were conducted at α=.05 significance level.

## 4 Results

The following results characterise the observed behaviours, using statistical metrics to describe patterns of engagement and attention distribution within the multi-robot setting.

### 4.1 Attention allocation and engagement structure

The aggregated descriptive statistics in Table 3 indicate that participants spent the majority of session time attending to task-relevant elements: the mean engagement (task-gaze) ratio was 0.88 (SD = 0.06; N = 16), consistent with sustained cognitive focus on the objects and robots throughout the interaction. Positive affect was present but more variable across participants (mean = 0.18, SD = 0.22), suggesting heterogeneous affective responses to the multi-robot configuration. Behavioural engagement, operationalised as the count of posture changes, was moderate-to-high on average (mean = 14.63, SD = 9.65), compatible with active, embodied participation in the task. While there is no established baseline for this specific metric in multi-robot interaction settings, observable body movements and posture shifts are commonly interpreted as indicators of behavioural engagement in educational and interaction research. Behavioural engagement is frequently operationalised through visible actions such as bodily movement, posture adjustments, and orientation toward interaction partners or task-relevant objects, reflecting active participation in the activity rather than passive observation Gomes et al. (2023), Booren et al. (2012).

**TABLE 3:** Aggregated children’s average engagement and attention during the sessions.

Task-gaze ratio	Positive affect ratio	Posture changes	Trajectories	Max trajectories
0.88 ± 0.06	0.18 ± 0.22	14.63 ± 9.65	10.54 ± 7.70	30.88 ± 15.24

*Engagement Ratio* refers to the cognitive engagement measure estimating the portion of time children gazed at either the robots or the objects. *Trajectories* refers to the length of gaze switches trajectories, in which the child continuously looks towards either Pepper, NAO or any of the objects.

Attention dynamics further reveal a quite variable level of attentional switching: the mean number of gaze trajectories per session was 10.54 (SD = 7.70), with an average maximum trajectory length of 30.88 (SD = 15.24). The relatively large standard deviations and high maxima indicate notable inter-individual variability in attentional mobility, motivating the use of non-parametric statistics and individual-level correlational analyses reported below. In particular, this variability justifies examining whether attentional complexity is related to gaze allocation (objects vs. robots) and whether affective or behavioural markers predict task-focused attention, as evaluated in the subsequent sections.

To characterise attention distribution, we compared gaze directed at the objects with gaze directed at the robots. As shown in Table 4, children spent significantly more time looking at the objects ( W=28 , p=.039 ). This indicates that the presence of two social agents did not shift focus away from the core learning task.

**TABLE 4:** Summary of Wilcoxon signed-rank tests for paired comparisons, focusing on children’s gazes towards objects and either of the robots.

Comparison	W	Z	p	Effect direction
Objects vs. Robots (gaze ratio)	28	–	0.039	Objects > robots

Next, we explored whether attention complexity, formalised as the number of transitions across gaze trajectories, was associated with attentional focus. Complexity showed a moderate, non-significant association with gaze directed at the robots ( ρ=.424 , p=.104 ) and a negligible relationship with gaze directed at the objects ( ρ=.071 , p=.797 ). Descriptively, this suggests that children who shifted attention more frequently tended to allocate slightly more attention to the robots, consistent with a pattern of social monitoring where the child actively checks the agents’ reactions between task actions.

### 4.2 Task performance and engagement by testing modality

Training performance was quantified as the ratio of correctly identified named objects to the total number of objects. Testing performance was measured as the ratio of correctly selected objects to the total number of objects. Results are shown in Table 5. Training performance suggests that children managed to follow the robot’s discourse and instructions, picking up the correct objects and, in most cases, repeating correctly the name associated with it. Testing performance was overall below chance when considering all sessions together. However, when only the pointing modality was considered, performance was slightly above chance, although still low. The very high standard deviation also shows that children had quite diverse performance, with some naming at least 2 of them correctly and one even all three. It is worth noting that both robots’ English text-to-speech uses an American English accent, and some words might not always be immediately understandable for some of the children (especially in the case where English was not the primary or only language spoken at home). This was also noted by some of the British-English native-speaking parents who engaged with the robot after the sessions. In fact, they commented that the children might not be used to hearing certain words pronounced a bit differently and, therefore, this might have impacted their response time or their general ability to follow the robot’s commands. Moreover, while the parents had no problems understanding the robot’s words, the robot’s speech recognition module struggled with understanding the British-English accent, preventing a fluid conversation. This was not part of the experimental session, but was still an interesting event which raised a very relevant issue which could not be addressed before the experiment (as the robot’s speech-to-text did not offer other choices of English accents and could not be changed), but that will be considered in future studies. Overall, the observations suggest that the testing modality did not systematically alter the nature of the interaction or engagement levels in this exploratory sample. It is important to stress that, given the very limited number of participants, these results do not seek to provide a conclusive assessment of the approach with respect to these considered measures. Here, we intend to describe the preliminary observed trends, pointing out possible interpretations, to be taken as a starting point for a full-scale user study.

**TABLE 5:** Performance results obtained for training and testing phase.

Modality	Testing	Training
Overall	0.719 ± 0.241	0.313 ± 0.333
Naming	0.722 ± 0.202	0.222 ± 0.272
Pointing	0.717 ± 0.273	0.367 ± 0.367

### 4.3 External correlates of engagement and performance

We first explored whether baseline language ability assessed with the QUILS language screener test (see Table 6) was associated with learning performance. Language scores were unrelated to performance during training (Spearman ρ=−.078 , p=.774 ). However, unexpectedly, baseline language ability showed a significant negative association with test performance (Spearman ρ=.657 , p=.006 ).

**TABLE 6:** Language screening scores by participant.

ID	Vocabulary	Syntax	Process	Overall
1	55.2	59.1	57.8	72.1
2	94.7	99.9	92.4	96.6
3	96.1	90.3	99.9	99.4
4	43.5	68.7	83.2	65.8
5	83.2	36.6	71.0	64.4
6	55.2	34.4	64.3	62.5
7	35.1	6.5	50.6	32.4
8	67.2	30.5	61.8	51.4
9	67.2	58.0	71.0	65.8
10	99.9	99.9	92.4	97.9
11	96.1	68.8	99.9	97.5
12	89.0	90.3	80.5	93.3
13	61.7	81.8	80.5	81.9
14	73.4	76.0	71.4	81.9
15	27.3	6.5	26.6	21.3
16	34.4	77.9	71.0	55.5

Finally, we examined age to assess potential developmental differences within the sample. Neither age nor number of siblings (see Table 1) correlated significantly with either performance (age–test score: ρ=.188 , p=.484 ; siblings–test score: ρ=.401 , p=.124 ) or robot gaze (age–robot gaze: ρ=−.260 , p=.331 ; siblings–robot gaze: ρ=−.044 , p=.870 ). This suggests that within the narrow age range of this sample (4–5 years), the multi-robot setup elicited comparable behavioural responses, supporting the feasibility of the design across this developmental span.

## 5 Discussion

This study presents an exploratory feasibility investigation into whether young children’s attentional, affective, and engagement behaviours can be effectively characterised within a multi-robot learning scenario in which two physically distinct robots assume complementary roles. Because of the limited sample size, regression analyses were deliberately avoided to reduce the risk of overfitting. Consequently, the quantitative findings reported here should be interpreted as descriptive associations that characterise the feasibility of the interaction, rather than as confirmatory evidence of treatment effects. Importantly, the aim of the present study was not to estimate population-level learning effects, but to examine whether a multi-robot configuration could be implemented successfully with young children and whether it would elicit structured, interpretable patterns of attention, affect, and engagement. Although each child was exposed to a small number of novel words, each session yielded a rich set of behavioural observations across time, allowing us to characterise how children organised their attention across objects and social agents within a multi-robot learning context. It is important to acknowledge that the recruitment for this study was severely constrained by the COVID-19 pandemic, as schools were resistant to external research involving novel technologies. This constraint motivated a feasibility-oriented design, prioritising the stability of the multi-robot setup and the collection of high-resolution behavioural data over statistical power for confirmatory hypothesis testing. This small sample size and the narrow age range (4–5 years) represent primary limitations of the current study, potentially impacting the generalisability of the results and the ability to detect subtle developmental effects. However, as an exploratory feasibility study, these data serve as a vital proof-of-concept for distributing pedagogical roles across two robots. The findings provide initial evidence that such a configuration is not only feasible but also capable of eliciting structured patterns of behaviour that can inform future investigations into multi-robot educational interactions.

In relation to RQ1, which examined how children distribute their attention and engagement during a word-learning task involving a tutor and a peer robot, the results indicate sustained engagement and a clear predominance of task-oriented attention across the sample. Across the sample, children showed sustained engagement and a clear predominance of task-oriented attention. Despite the presence of two social agents, children reliably directed their gaze toward the objects central to the word-learning task, suggesting that the social dynamics introduced by the tutor–peer configuration did not detract from task focus. This is notable given prior work reporting that highly sociable robots can, in some cases, dampen learning performance among younger children (Kennedy et al., 2015a; Herberg et al., 2015). This pattern is notable given prior work reporting that highly sociable robots can, in some cases, dampen learning performance or distract attention among younger children (Kennedy et al., 2015a; Herberg et al., 2015). The present findings suggest that social presence alone is not sufficient to disrupt task focus, but rather, the distribution of pedagogical and social roles across agents may be critical for maintaining a balance between social engagement and task relevance. By separating instructional and peer-like functions across two physically distinct robots, the interaction preserved task-oriented attention while still enabling social engagement, potentially mitigating the trade-offs identified in earlier single-robot studies.

This is notable given prior work reporting that highly sociable robots can, in some cases, dampen learning performance among younger children (Kennedy et al., 2015a; Herberg et al., 2015). Here, the combined use of two role-differentiated robots did not appear to impose such a cost, indicating that complementary roles may help maintain a balance between social interaction and task relevance. These findings directly address RQ1(a), confirming that children can maintain task-oriented gaze even in a complex multi-robot environment. Furthermore, the observation that children with more complex gaze trajectories allocated slightly more attention to the robots provides insight into RQ1(b) regarding the relationship between attentional complexity and social focus.

Further addressing RQ1, the exploratory analyses revealed trends linking attentional complexity, gaze allocation, and affective responses toward the robots and task. Children who displayed more complex patterns of attentional shifting tended to allocate slightly more attention to the robots, raising the possibility that the multi-agent context may introduce additional social or exploratory demands for some learners, as also observed in some studies in the literature (Dong et al., 2024). This observation aligns with the tripartite framework of engagement—affective, cognitive, and behavioural—proposed by Leite et al. (2014), Maggi et al. (2025). By adopting this framework, we could capture the nuance of how children navigate social scaffolding provided by multiple agents (Efthymiou et al., 2020). While the small sample size and limited word set warrant a cautious interpretation of statistical outcomes, the significant variation in individual children’s results provides a preliminary basis for addressing RQ1(c) regarding the behavioural predictors of affect. Specifically, the data show that high task-engagement did not consistently result in high positive affect (see Table 3), suggesting that task-oriented gaze duration alone may not be a consistent predictor of a child’s affective state in this multi-robot setting.

Addressing RQ2, which examined how individual characteristics relate to children’s task engagement and performance, the results revealed a mixed pattern of associations. Although age, household composition, and media exposure were unrelated to performance or engagement in this sample, baseline language ability showed a robust negative association with recall. This suggests that children with higher baseline language scores performed worse on the recall test, an unexpected pattern that may reflect task-specific strategy differences, ceiling effects, or sampling variability. Another possible explanation is that the proposed setting did not sufficiently engage children with higher baseline language scores, who may have found the task less challenging or motivating. This result problematizes the “one-size-fits-all” approach to multi-robot interaction, suggesting it may inadvertently disengage advanced learners. Furthermore, the overall low test performance despite high engagement problematizes the assumption that social presence naturally facilitates learning. Technical factors, such as the robots’ American English accents, may have restricted the effectiveness of the interaction for some children, highlighting how basic technical limitations can undermine complex social scaffolding. In the context of RQ2, the inclusion of age as a variable was intended to detect developmental shifts in engagement. The lack of significant differences between 4- and 5-year-olds suggests that the interaction design is robust across this age gap. While factors such as age and media exposure showed limited influence, the negative association with baseline language ability highlights the complexity of how individual learner characteristics relate to performance in multi-agent systems.

Overall, the results indicate that the multi-robot configuration is feasible for studying children’s behaviour during learning and can be implemented in a way that preserves engagement and task focus. The quantitative data serve to back the qualitative observation that children were able to accept the two robots in distinct roles without confusion. The findings illustrate the value of using fine-grained behavioural measures, including gaze patterns, affective cues, and engagement markers, to capture how children respond to distinct robotic roles embedded within a shared learning activity. The structured behavioural patterns observed here provide a foundation upon which more controlled, hypothesis-driven studies can build and highlight the potential of multi-robot approaches to deepen our understanding of how children engage with multiple social partners during learning and how such interactions may be harnessed to support educational development. In particular, future work may investigate how tutor and peer roles interact over time, whether role-switching or adaptive role behaviours influence learning, how children coordinate their attention when navigating contributions from multiple embodied agents, and whether the presence of multiple robots offers measurable advantages over single-robot configurations. Beyond demonstrating feasibility, it is important to consider whether multi-robot configurations ultimately expand or restrict the practical application of educational robotics. On one hand, distributing roles may expand pedagogical possibilities by allowing for more complex social scaffolding. On the other hand, the requirement for multiple synchronised units may restrict use in standard educational settings due to increased cost, technical complexity, and the potential for higher cognitive load. Future research should evaluate with a larger sample whether the social and engagement benefits of adding a second “novice” agent justify these significant resource requirements. Relatively to the current work, another limitation that may have had an impact on the interaction is the slow pace of the interaction (which by design was not too fast to allow children to follow the interaction without smoothly), which for some children may have made it less engaging; similarly, the choice of the objects might have simplified too much the task, as only one of the objects in every trial was unknown, while the rest were every day familiar items. This might as well have rendered the interaction too predictable. Regarding the technological limitations, instead, the two main limitations are the inability of the robots to manipulate elements in the scene, therefore having to rely exclusively on the gaze direction, and the use of two external cameras mounted in the experimental setting, which may have drawn the attention of the participants, distracting the children (even if marginally). All these limitations will be addressed in future extensions of this study.

## 6 Conclusion

This study examined the feasibility of deploying two physically distinct robots, framed in complementary pedagogical roles, within a single early word-learning activity. The results indicate that such a multi-robot configuration can be implemented with young children in a way that preserves engagement and supports sustained task-oriented attention. Across the interaction, children predominantly focused on task-relevant objects, and their behavioural responses were structured and measurable, suggesting that they were able to coherently navigate the presence of multiple embodied social agents.

At the same time, these findings warrant a cautious interpretation. While the observed focus on learning objects is consistent with the interpretation that the tutor–peer configuration did not distract from the task—in contrast to concerns raised in some prior work—an alternative explanation is that the robots may have exerted a relatively limited influence on the learning process itself. From this perspective, sustained object-directed attention may reflect effective task framing rather than a direct benefit of the multi-robot social dynamics. The present study, therefore, does not allow strong claims about learning advantages attributable to the presence of two robots, but instead constrains the space of plausible effects by showing that multi-robot interactions need not inherently disrupt attention or engagement.

As an exploratory proof-of-concept, these findings are best understood as foundational rather than confirmatory. The small sample size and narrow age range (4–5 years) limit generalisability and preclude fine-grained analysis of developmental trends or individual differences. Future research should build on this work by employing larger and more diverse samples, richer learning assessments, and comparative designs, such as contrasting single-robot and multi-robot interactions or introducing adaptive role-switching. Such studies will be essential for determining not only whether multi-robot configurations are feasible, but under what conditions distinct robotic roles meaningfully shape children’s attention, engagement, and learning over time.

## Data Availability

The original contributions presented in the study are included in the article/supplementary material, further inquiries can be directed to the corresponding author.

## Footnotes

1
https://us.softbankrobotics.com/pepper

2
https://us.softbankrobotics.com/nao

3
https://osf.io/nbrhk

4
http://www.sussex.ac.uk/wordlab/noun

5 United Kingdom’s national curriculum stages

6
https://www.ntnu.edu/e-ladda/e-ladda

7
https://quilscreener.com

8 ELAN (Version 7.0) [Computer software]. (2025). Nijmegen: Max Planck Institute for Psycholinguistics, The Language Archive. Retrieved from https://archive.mpi.nl/tla/elan
